# Conformational Differences between Active Angiotensins and Their Inactive Precursors

**Published:** 2012

**Authors:** O.N. Solopova, L.P. Pozdnyakova, N.E. Varlamov, M.N. Bokov, E.V. Morozkina, Т.А. Yagudin, P.G. Sveshnikov

**Affiliations:** Russian Research Center for Molecular Diagnostics and Therapy; Bach Institute of Biochemistry, Russian Academy of Sciences

**Keywords:** peptides, conformation, angiotensins, angiotensinogen, monoclonal antibodies

## Abstract

The peptide conformation in the context of a protein polypeptide chain is influenced by proximal amino acid residues. However, the mechanisms of this interference remain poorly understood. We studied the conformation of angiotensins 1, 2 and 3, which are produced naturally in a sequential fashion from a precursor protein angiotensinogen and contain an identical peptide core structure. Using the example of angiotensins 1, 2 and 3, it was shown that similar amino acid sequences may have significant conformational differences in various molecules. In order to assess the conformational changes, we developed a panel of high-affinity mouse monoclonal antibodies against angiotensins 1, 2 and 3 and studied their cross-reactivity in indirect and competitive ELISAs. It was found that the conformations of inactive angiotensin1 and the corresponding fragment of angiotensinogen are similar; the same is true for the conformations of active angiotensins 2 and 3, whereas the conformations of homologous fragments in the active and inactive angiotensins differ significantly.

## INTRODUCTION

Since the appearance of the hybridoma technology in 1975 [1], a large number of monoclonal antibodies to various substances have been obtained. Despite this, new antibodies are still highly sought; namely, antibodies with particular properties, antibodies to particular epitopes and to newly discovered proteins and other organic and inorganic compounds. Normally, it is possible to obtain new proteins only in limited amounts, and frequently it is very difficult or even impossible to isolate them in their pure form with the natural conformation preserved. These kinds of proteins cannot be used for immunization to obtain antibodies; hence, in the majority of cases, the only viable option is immunization with synthetic peptides corresponding to particular fragments of the desired protein. Along with the apparent advantages, this approach also has a number of shortcomings: *i.e.* , peptides, when found in proteins, have a significantly lower degree of freedom than when they are in a free state. As a result, the antibodies against peptides are not always capable of binding to full-size proteins [2].

The oft-cited example of the structural differences between peptides found in their protein precursor and peptides in a free state is human angiotensinogen and its metabolites; *i.e.* , angiotensins 1, 2 and 3. Angiotensin 1 (Ang1) is a prohormone that consists of 10 amino acid residues and is produced from angiotensinogen as a result of the cleavage of the N-terminal peptide [3]. The Ang1 exhibits no physiological activity and plays the role of a substrate in the formation of angiotensins 2 and 3. Angiotensin 2 (Ang2) differs from angiotensin 1 by the absence of two C-terminal amino acid residues. Angiotensin 3 (Ang3) is shorter than angiotensin 2 by one N-terminal residue ( *Figure* ). Ang1 contains the same amino acids as Ang2; however, Ang1 is incapable of binding to the receptors of Ang2 and thereby cannot initiate effector functions [4]. The most likely causal factor behind this phenomenon is the conformational differences between angiotensins 1 and 2. To confirm this hypothesis, we obtained monoclonal antibodies against angiotensins 1, 2 and 3 and studied their cross-reactivity to various angiotensins and angiotensinogen.

## EXPERIMENTAL

**Table:** Amino acid sequences of the precursors of angiotensin 2 and its metabolites.

	1	2	3	4	5	6	7	8	9	10	11	12	13	14
Angiotensinogen	Asp -	Arg -	Val -	Tyr -	Ile -	His -	Pro -	Phe -	His -	Leu -	Leu -	Val -	Tyr -	Ser
Angiotensin 1	Asp -	Arg -	Val -	Tyr -	Ile -	His -	Pro -	Phe -	His -	Leu				
Angiotensin 2	Asp -	Arg -	Val -	Tyr -	Ile -	His -	Pro -	Phe						
Angiotensin 3	-	Arg -	Val -	Tyr -	Ile -	His -	Pro -	Phe						

In this work human recombinant angiotensinogen (“Sigma”, USA), angiotensins 1, 2 and 3 (“American Peptide”, USA), recombinant Hsp70 from *Mycobacterium tuberculosis* obtained in our laboratory [5], BALB/c mice, and the mouse myeloma cell line Sp2/0 were used.

**The Production of Monoclonal Antibodies against Angiotensins 1, 2, and 3**

Mice were immunized in their hind paws with angiotensins conjugated with Hsp70: an adjuvant protein from *M. tuberculosis* , as described in [6]. The procedure was carried out twice with an interval of two weeks with a dosage of 100 µg of the conjugate per one immunization. The first immunization was performed using Freund’s complete adjuvant and the second through Freund’s incomplete adjuvant. On the third day following the second immunization, popliteal lymph node cells were hybridized with sp2/0 myeloma cells in accordance with the standard procedure [1]. The supernatants of the hybrids were tested by means of indirect [7] and competitive enzyme-linked immunosorbent assays (ELISAs) [8]; positive clones were cloned 2–4 times, monoclonal antibodies were produced in ascitic fluids of mice and were isolated by affine chromatography on protein G-sepharose [9]. The purity of the antibodies was controlled by electrophoresis in 12% polyacrylamide gel as described in [10].

**Characterization of Monoclonal Antibodies Produced**

The specificity of the obtained antibodies was determined by means of indirect and competitive ELISAs [7, 8]. The affinity of the antibodies against each target was assessed via the measurement of the dissociation constant ( *K*_d_ ) as described in [11] by Klotz, with modifications made by Friguet [12].

## RESULTS AND DISCUSSION

The human and mouse angiotensins 1, as well as angiotensins 2 and 3, have identical amino acid sequences [13]. In addition, angiotensins 2 and 3 exhibit physiological activity and their introduction into the body at doses needed for immunization (10–50 µg/mouse) leads to a fatal outcome even in the case of intramuscular and subcutaneous injections. Altogether, it makes angiotensins extremely inconvenient immunogens; however, their conjugation with Hsp70, an adjuvant protein from *M. tuberculosis, * allowed to overcome the immunological tolerance and to eliminate the toxicity. As a result, we obtained monoclonal antibodies against each angiotensin.

The specificity of each antibody produced was determined by enzyme-linked immunosorbent assay ( *Table 1* ). Indirect ELISA revealed interaction between the antibodies and the sorbed targets. In the aforementioned system, part of the structural units of angiotensinogen and peptides is found inaccessible to antibodies and the other part is distorted. Through the competitive ELISA, we established the interaction of the antibodies with the protein and peptides in the single-phase system, *i.e.* , a solution; in turn, the determination of the dissociation constants allowed us to quantitatively estimate the interaction force ( *Table 2* ).

The most affine antibody obtained against angiotensin 1, AngC11 ( *K*_d_  = 1.3 × 10 ^–10^ ), almost does not bind to sorbed Ang1 and also interacts with angiotensins 2 and 3 in neither indirect nor competitive ELISA. At the same time, AngC11 recognizes angiotensinogen both in the sorbed form and in solution. All these factors indicate that either the epitope of this antibody contains amino acid residues that detach when angiotensins 2 and 3 are formed, or that the structures of this fragment in Ang1, Ang2, and Ang3 differ to the extent that the antibody is only capable of binding to Ang1.

**Table 1:** Interaction of antibodies with angiotensins 1, 2, and 3 and with angiotensinogen in indirect and competitive ELISAs

Immunogen	Antibody	Indirect ELISA	Competitive ELISA
A-gen	А1	А2	А3	A-gen	А1	А2	А3
Аng 1	AngE9	-	+	-	-	-	+	-	-
	AngC9	+	+	-	-	+	+	±	±
AngC11	+	±	-	-	+	+	-	-
Аng 2	AngIIE7	-	-	+	±	-	-	+	+
Аng 3	AngIII B7	-	-	+	±	n.d.	n.d.	n.d.	n.d.
AngIII F7	-	-	+	±	n.d.	n.d.	n.d.	n.d.

In contrast, the antibodies obtained as a result of the immunization with angiotensins 2 and 3 recognize only angiotensins 2 and 3, without making any difference between them in competitive analysis and preferring Ang2 in indirect ELISA, regardless of which angiotensin immunization (second or third) is performed. The fact that angiotensin 2 was better recognized in indirect ELISA can be explained simply by its superior ability to sorb Ang2 than the shorter, less hydrophilic Ang3. None of the antibodies against Ang2 and Ang3 recognizes angiotensin 1 and the angiotensinogen in any mode of enzyme-linked immunosorbent assay. Despite this, Ang1 and angiotensinogen containing amino acid sequences are also found in the structures of angiotensins 2 and 3.

## CONCLUSIONS

**Table 2:** Dissociation constants ( *K*_d_ ) of monoclonal antibodies against angiotensins 1 and 2 with different targets

Antibody	Kd, M			
	A-gen	Аng 1	Аng 2	Аng 3
AngE9	>10^-5^	4.7х10^-7^	>10^-5^	>10^-5^
AngC9	4.0х10^-8^	7.7х10^-9^	3.0х10^-5^	3.0х10^-5^
AngC11	1.25х10^-8^	1.3х10^-10^	5.5х10^-6^	2.3х10^-5^
AngIIE7	>10^-5^	>10^-5^	6.0х10^-7^	2.0х10^-6^

Summarizing the results, we can draw the following conclusions:

1. Angiotensin 1 in free state and in angiotensinogen has the same conformation;

2. The detachment of two amino acid residues from Ang1 changes the conformational structure of the entire peptide; the produced angiotensin 2 has a different conformation than the conformation of the identical fragments in angiotensin 1 and angiotensinogen;

3. The detachment of one amino acid residue from angiotensin 2 does not significantly alter the conformational structure of the peptide; the conformation of angiotensin 3 produced as a result is similar to the conformation of angiotensin 2 and differs completely from the conformation of the corresponding fragments in Ang1 and angiotensinogen; 

4. When short peptides are used for producing monoclonal antibodies against proteins, the probability of a complete transformation of the protein antigenic determinants synthesized in the form of peptides should be taken into account; peptides sorbed in solid phase can also have significant conformational differences from dissolved peptides with the same amino acid sequence.

The transformation of inactive angiotensin 1 and angiotensinogen into their active forms, Ang2 and Ang3, is accompanied by a significant conformational rearrangement of the corresponding fragments in peptide and protein molecules. 

## Abbreviations

Ang1: human angiotensin 1
Ang2: human angiotensin 2
Ang3: human angiotensin 3
ELISA: enzyme-linked immunosorbent assay
PAG: polyacrylamide gel
Hsp70: heat shock protein with a molecular mass of 70 kDa
*K*_d_: dissociation constant
